# High prevalence of cardiovascular risk factors in Peruvian adolescents living in a peri-urban shantytown: a cross-sectional study

**DOI:** 10.1186/s41043-017-0093-1

**Published:** 2017-05-22

**Authors:** Elizabeth S. Abbs, José Viñoles, Jorge O. Alarcón, Heather M. Johnson, Joseph R. Zunt

**Affiliations:** 1 Department of Medicine, University of California San Francisco, 505 Parnassus Ave, San Francisco, CA 94143 USA; 2 Instituto de Medicina Tropical “D.A. Carrion” de la Universidad Nacional Mayor de San Marcos, Jiron Jose Santos Chocano 199, Lima, Peru; 3 Division of Cardiovascular Medicine, University of Wisconsin School of Medicine and Public Health, H4/512 CSC, MC 3248, 600 Highland Avenue, Madison, 53792 WI USA; 4 Departments of Neurology, Global Health and Medicine (Infectious Disease) of University of Washington, 325 Ninth Avenue, Seattle, WA USA

**Keywords:** Cardiovascular disease, Primary prevention, Risk factors, Adolescent health, Peru

## Abstract

**Background:**

Adults of the peri-urban Peruvian shantytown of Lomas de Zapallal have a high prevalence of risk factors for developing cardiovascular disease (CVD)—likely due to behavioral choices established during childhood and adolescence. To guide the development of community-based risk reduction programs, we assessed the prevalence of risk factors for developing CVD among adolescents.

**Methods:**

We collected cross sectional data from adolescents of Peruvian peri-urban shantytown to evaluate four domains of CVD risk factors: (1) clinical (blood pressure, fasting blood glucose, and blood lipids), (2) anthropometric (height, weight, and waist circumference), (3) behavioral (physical activity, diet, and substance abuse), and (4) psychosocial (mental health and violence).

**Results:**

We enrolled 275 adolescents (56.4% female, mean age 14 years). Prevalence of overweight or obese status was 27.8%. High blood pressure was more common in males (37.4%) than females (20.5%) (*p* = 0.002). Total cholesterol was elevated (>170 mg/dL) in nearly half (45.5%) of the adolescents, and 71% had impaired fasting blood glucose (>100 mg/dL). Females were less likely to exercise daily (95.4%) than males (84.2%) (*p* = 0.002) but reported higher rates of depression (66.4%), anhedonia (67.6%), and self-harm behavior (37.9%) (all *p* < 0.01).

**Conclusions:**

Adolescents living in the peri-urban population of Puente Piedra had high prevalence of risk factors for future development of CVD; preventative efforts focused on improving nutrition, increasing physical inactivity, and addressing mental health conditions could reduce such risk factors.

## Background

Cardiovascular disease (CVD) is a major cause of mortality and morbidity throughout the world [1, 2]. Obesity, hypertension, abnormal blood glucose, physical inactivity, poor diet, substance abuse, depression, and self-harm increase the probability of developing CVD [3–6]. These risk factors disproportionately affect low-income populations, thereby increasing their risk for developing CVD and other chronic diseases [7–9]. In Peru, a survey of adult residents living in the Northern Lima shantytown of Lomas de Zapallal revealed a high prevalence of risk factors for CVD, including obesity (53%), hypertension (15%), tobacco use (34% in males), binge alcohol use (35% in males), and depression (12%) [10].

Early detection of risk factors may offer opportunities for lifestyle change to reduce morbidity and mortality [11]. Adolescence is a formative time when lifestyle choices (e.g., substance use, dietary choices, and physical activity) are observed, modeled, and reinforced into life-long practice [12]. Persistence of poor lifestyle choices during and after adolescence heightens the risk for obesity, atherosclerosis [13–18], and mental illness as adults. Psychiatric disorders (especially depression) that manifest during adolescence independently predict early development of CVD and are associated with increased mortality [6, 19, 20].

Few studies have examined risk factors for developing CVD in shantytown populations, and even fewer have examined adolescents living in low-income settings [21–24]. To guide CVD prevention efforts, we conducted a cross-sectional study to assess the prevalence of obesity, high blood pressure, cholesterol, fasting blood sugar, physical activity, diet, depression, and trauma in school-aged adolescents attending Colegio Pitagoras 8183 in Lomas de Zapallal, Puente Piedra, Lima, Peru.

## Methods

### Setting

Lomas de Zapallal is a small *pueblo joven* (“shantytown”) community in the Northern Lima district of Puente Piedra, Peru. The majority of houses in the area are made of cement with corrugated metal roofs. A sanitation system was installed in 2009 that provides inconsistent portable water to 90% of inhabitants [10, 25]. Many adults in Lomas de Zapallal work several jobs, often traveling up to 5 h daily by public transport to reach their places of employment. The closest health post is a 15-min bus ride, but patients often wait several hours for medical attention. The district has few schools, the largest being a public school named Colegio Pitágoras 8183 with approximately 1500 primary and secondary school students (based on community discussions, 2015). As the result of a collaboration between Universidad Nacional Mayor de San Marcos and University of Washington, Colegio Pitágoras 8183 has participated in various public health studies since 2009 [25]. All parts of the present study were conducted on the Colegio Pitágoras 8183 campus.

### Study design

A cross-sectional study was conducted between November 2015 and April 2016 to evaluate the prevalence of risk factors for CVD: anthropometric and clinical (weight, height, BMI, abdominal circumference, blood pressure, capillary puncture total cholesterol, hemoglobin, and fasting blood glucose), as well as behavioral and psychosocial (diet, physical activity, mental health, stress, and drug use). Approval was obtained from the Institutional Review Board of the Universidad Nacional Mayor de San Marcos. Exempt status was granted from the Institutional Review Board of the University of Washington as the present study did not meet their definition of research.

### Participants

All adolescents in second, third, and fourth grade of secondary school at Colegio Pitágoras 8183 were invited to participate. Student grade level was classified by their 2015 status (first to fourth grade). Only students with parental consent and who provided voluntary informed assent were enrolled.

### Data sources

A team of five trained Peruvian health students and professionals obtained anthropometric and clinical data. Data was collected at four stations: (1) weight and height to calculate BMI and overweight/obesity status, (2) waist circumference (WC), (3) blood pressure (BP), and (4) capillary blood draw. Students were instructed to remove their shoes, hair buns, and jackets prior to measuring weight and height. Two nurses drew capillary blood for three point-of-care (POC) assays: total cholesterol (Accutrend Plus: Roche Diagnostics, Switzerland), fasting glucose (AcuChek: Roche Diagnostics, Switzerland), and hemoglobin (Hemocue Hb 201: Quest Diagnostics, Sweden).

BP was measured using an automated sphygmomanometer (Citizen: Veridian Healthcare, USA) with an appropriately sized cuff on the participant’s left upper arm, per guidelines and study protocol [26]. If readings were >130/90 mmHg, participants were asked to rest 5 min before a repeat measurement was obtained from each arm. The lowest reading was documented. Percentiles for body mass index (BMI), systolic blood pressure, and diastolic blood pressure were calculated utilizing participant age, gender, and height criteria [27, 28]. We utilized adolescent-specific scales and derived height percentiles from Peruvian NIH and Ministry of Health [29]. Criteria for abnormal values are described in the footnotes of Table 1. Behavioral and psychosocial data were collected via a four-page questionnaire. We administrated Spanish-language validated questionnaires based on the WHO STEPs survey, the Center for Disease Control (CDC) Youth Risk Behavioral Surveillance System (YRBSS), and the National Health and Nutrition Examination Survey (NHANES) to allow comparison with other populations. Risk factors for mental health and violence were assessed by Spanish-language validated versions of the Patient Health Questionnaire (PHQ-2) and Adverse Childhood Experience (ACE) survey [11, 30–33]. We adapted American Heart Association’s Healthy Heart criteria to define adequate physical activity through self-reported daily exercise; inadequate physical activity was defined as exercising less than five times per week. Dietary ideals were defined using the same criteria and measured daily consumption of fruit, vegetables, and frequency of adding table salt to meals or consuming processed food [2]. Prevalence of depression and anhedonia were defined by participant’s answers to the PHQ-2, while prevalence of self-harm, family history of mental health, and violence were defined by positive responses to the ACE survey.

**Table 1:** Sex-specific demographic, clinical, and behavioral risk factor prevalence in adolescents of a Shantytown in Lima, Peru

Variable	Total		Male		Female		*p* value
	*N*	%, (95% CI)	*N*	%, (95% CI)	*N*	%, (95% CI)	
Demographics							
Gender	275		120	43.6, (37.9, 49.6)	155	56.4, (50.4, 62.2)	
Age (years)	275	(*μ*, *SD*) 14, 1	120	14, 1	155	14, 1	
2015 school grade	275		120		155		
First	74	26.9, (22.0, 32.5)	37	30.8, (23.1, 39.8)	37	23.9, (17.8, 31.3)	
Second	73	26.5, (21.6, 32.1)	27	22.5, (15.8, 31.0)	46	29.7, (23.0, 37.4)	
Third	62	22.5, (18.0, 27.9)	32	26.7, (19.4, 35.4)	30	19.4, (13.8, 26.4)	
Fourth	66	24.0, (19.3, 29.4)	24	20.0, (13.7, 28.2)	42	27.1, (20.6, 34.7)	
Household population	269	(*μ*, *SD*) 5, 2	116	5, 3	153	5, 2	
Health visit	262	38.2, (32.5, 44.2)	111	31.5, (23.5, 40.9)	151	43.0, (35.3, 51.1)	
Family history (FH)^a^	273		118		155		
Diabetes (DM)		16.9, (12.8, 21.8)		15.3, (9.8, 23.1)		18.1, (12.7, 25.0)	
Hypertension (HTN)		13.6, (10.0, 18.2)		13.6, (8.4, 21.1)		13.6, (9.0, 20.0)	
High cholesterol		23.2, (18.5, 28.6)		22.9, (16.1, 31.5)		23.4 (17.3, 30.8)	
Heart disease		9.9, (6.9, 14.1)		7.6, (4.0, 14.1)		11.6 (7.4, 17.8)	
Clinical risk factors							
Nutritional status^b^	266		115		151		0.78
Normal weight		72.2, (66.5, 77.3)		71.3, (62.2, 78.9)		72.9, (65.1, 79.4)	
Overweight, obese		27.8, (22.7, 33.5)		28.7, (21.1, 37.8)		27.2, (20.6, 34.9)	
Abdominal obesity^c^	266	24.8, (20.0, 30.4)	115	26.1, (18.8, 35.0)	151	23.8, (17.7, 31.4)	0.67
Hypertension^d^	266		115		151		0.002
Normal BP		72.2, (66.5, 77.3)		62.6, (53.3, 71.1)		79.5, (72.2, 85.2)	
Abnormal BP		27.8, (22.7, 33.6)		37.4, (28.9, 46.7)		20.5, (14.8, 27.8)	
Anemia (mild, mod)^c^	265	28.7, (23.5, 34.5)	114	23.7, (16.7, 32.5)	151	32.5, (25.4, 40.4)	0.12
Total Cholesterol^f^	266		115		151		0.57
Normal		54.5, (48.5, 60.4)		56.5, (47.2, 65.4)		53.0, (44.9, 60.9)	
Abnormal		45.5, (39.6, 51.6)		43.5, (34.6, 52.8)		47.0, (39.1, 55.1)	
FBG^g^	183		70		113		0.02
Normal		29.0, (22.8, 36.0)		22.9, (14.3, 34.4)		32.7, (24.6, 42.0)	
Abnormal		71.0, (64.0, 77.2)		77.1, (65.6, 85.7)		67.3, (58.0, 75.4)	
Behavioral risk factors							
Physical activity^h^	265	*%, (95% CI)*	114	*%, (95% CI)*	151	*%, (95% CI)*	0.002
Adequate		9.4, (6.4, 13.6)		15.8, (10.1, 23.8)		4.6, (2.2, 9.5)	
Inadequate		90.6, (86.4, 93.6)		84.2, (76.2, 89.9)		95.4, (90.5, 97.8)	
Sedentary behavior	265	*(μ, SD)*		*(μ, SD)*		(*μ, SD)*	
Television (hours/day)		3.4, 3.6		3.1, 4.2		3.4, 2.9	
Cell phone (hours/day)		3.9, 5.5		3.6, 5.3		3.1, 4.2	
Internet (hours/day)		2.1, 4.1		2.4, 3.8		2.2, 4.6	
Dietary^i^	274	*%, (95% CI)*	119	*%, (95% CI)*	155	*%, (95% CI)*	
Ideal fruit intake		42.3, (36.6, 48.3)		37.0, (28.7, 46.1)		53.6, (45.6, 61.3)	0.12
Poor fruit intake		57.7, (51.7, 63.4)		63.0, (53.9, 71.3)		46.5, (38.7, 54.4)	
Ideal veg. intake		32.1, (26.8, 37.9)		33.6, (25.6,4 2.7)		31.0, (61.2, 75.9)	0.64
Poor veg. intake		67.9 (62.1, 73.2)		66.4, (57.3, 74.4)		69.0, (61.2, 75.9)	
Frequent salt use	273	11.4, (8.1, 15.7)	118	8.5, (4.6, 15.2)	155	13.6, (9.0, 20.0)	0.19
Substance use^j^		*%, (95% CI)*		*%, (95% CI)*		*%, (95% CI)*	
Tobacco Use	257	16.3, (12.3, 21.4)	112	18.8, (12.5, 27.2)	145	14.5, (9.6, 21.3)	0.36
FH tobacco use	246	14.2, (10.4, 19.2)	107	13.1, (7.8, 21.0)	139	15.1, (10.0, 22.2)	0.65
Alcohol use	247	49.4, (43.2, 55.7)	110	50.0, (40.6, 59.4)	137	48.9, (40.5, 57.3)	0.86
FH alcohol abuse	245	17.1, (12.9, 22.4)	108	17.6, (11.4, 26.1)	137	16.8, (11.4, 24.1)	0.87
Marijuana use	253	4.3, (2.4, 7.7)	106	4.7, (1.9, 11.0)	147	4.1,(1.8, 8.9)	0.81
Mental health^k^		*%, (95% CI)*		*%, (95% CI)*		*%, (95% CI)*	
Depression	246	55.3, (49.0, 61.4)	106	40.6, (31.5, 50.3)	140	66.4, (58.1, 73.8)	<0.0001
Anhedonia	242	60.3, (54.0, 66.2)	100	50.0, (40.2, 59.8)	142	67.6, (59.4, 74.9)	0.006
FH mental illness	227	16.7, (12.4, 22.2)	99	8.1, (4.0, 15.5)	128	23.4, (16.8, 31.7)	0.002
Self-harm	248	28.2, (22.9, 34.2)	108	15.7, (9.9, 24.0)	140	37.9, (30.1, 46.3)	<0.0001
Violence^l^		*%, (95% CI)*		*%, (95% CI)*		*%, (95% CI)*	
Physical, home	253	28.9, (23.6, 34.8)	113	25.7, (18.4, 34.6)	140	31.4, (24.2, 39.7)	0.31
Physical, school	262	7.6, (5.0, 11.6)	115	10.4, (6.0, 17.6)	147	5.4, (2.7, 10.6)	0.13
Sexual	260	2.7, (1.3, 5.6)	113	0.9, (0.1, 6.2)	147	4.1, (1.8, 8.9)	0.11

^a^Majority of participants denied FH knowledge: 35.9% for DM, 54.6% for HTN, 39.3% for cholesterol, and 26.0% for heart disease answered “don’t know”

^b^Age and gender-specific percentiles used WHO guidelines for adolescents [14] to define: “Normal weight” as 5 < BMI% > 85, and “Abnormal weight” as 85 < BMI% > 95 (overweight) and BMI% > 95 (obese)

^c^Abdominal obesity defined as participants with “high” and “very high” future risk secondary to waist circumference as per national cut-offs from the Peruvian NIH and Ministry of Health [29]

^d^Peruvian NIH and Ministry of Health [29] defined “No anemia” as >12 g/dL for females and >13 g/dL for males, and “Anemia” as <11 g/dL for both genders

^e^Age, gender, and height-specific percentiles used NIH [50] guidelines for adolescents to define: “Normal BP” as 5 < BP% > 85, “Abnormal BP” as 85 < BP% > 95 (prehypertension), and BP% > 95 (hypertension I)

^f^AHA cardiovascular ideals for adolescents [2] defined cholesterol as “Normal” if <170 mg/dL and “Abnormal” if >170 mg/dL. POC machine reading of “lo” included as “normal”

^g^AHA cardiovascular ideal for adolescents [2] defined fasting blood glucose (FBG) as “Normal” if <100 mg/dL and “Abnormal” if >100 mg/dL. Only participants in fasting state included; 31% of 266 were excluded due to “non-fasting” status

^h^“Adequate” physical activity determined by daily intense physical activity and “Inadequate” as less than daily physical activity as per adolescent recommendations from AHA cardiovascular ideal [2], CDC YRBSS [11], and CDC NHANES [11, 30]

^i^As per AHA cardiovascular ideal [2], CDC YRBSS [11]; CDC NHANES [11, 30] adolescent recommendations, “Ideal” fruit and vegetable intake determined by daily intake (7 days/week) and “Poor” as less than daily; “Frequent salt intake” determined by endorsement of “often or always” putting condiments and salt on food at meals

^j^Substance use determined by endorsed lifetime use (one or more) with questions based on surveys from: Peruvian NIH and Ministry of Health [29], CDC Youth Risk Behavioral Surveillance System (YRBSS), and the National Health and Nutrition Examination Survey (NHANES) [11, 30–33]

^k^Rates of depression and anhedonia in the “last 2 weeks” defined by Spanish-language PHQ-2 [33]

^l^Physi cal and sexual violence are based on questions from the Spanish-language Adverse Childhood Experience (ACE ) questionnaire [31]

### Statistical analysis

Data were collected and managed using REDCap, a secure web-based electronic data capture tool, hosted at the University of Washington [34] and analyzed using STATA, version 14.1 (STATA Corp, Collegetown, TX). Descriptive statistical analysis via cross-tabulation and chi-square tests were used to evaluate associations between risk factors and gender. Univariate logistic regression was used to estimate the odds ratio (OR) and prevalence ratio (PR) with 95% confidence intervals (95% CIs) of the associations of gender with clinical outcomes, nutrition, physical activity, substance use, and mental health. Data were evaluated for cofounding effect from age, gender, grade, health status, and home population; no significant cofounders were identified.

## Results

### Study population

All adolescents attending Colegio Pitágoras 8183 in second, third, or fourth grade of secondary school during the 2015 or 2016 school years were invited to participate. Of the 275 participants enrolled, one male participant did not complete the behavioral questionnaire and nine participants (five males, four females) chose not to participate in the clinical data collection.

**Fig. 1:**
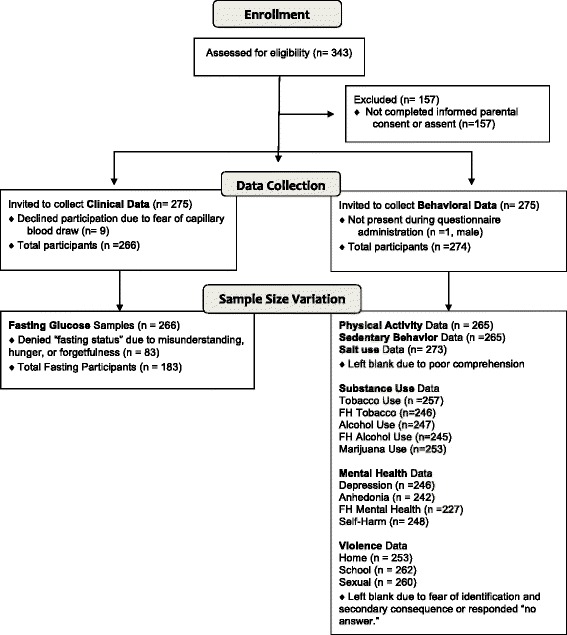
Flow diagram of study population enrollment, data collection and missing data

Of the 266 participants who completed the clinical data collection, 252 were given a numeric score for total cholesterol. Glucose samples were attained for all 266 participants; however, only 183 (68.8 and 60.8% males and 74.8% females) reported being in “fasting state.” Analysis of glucose data excluded participants who denied a fasting state. The process of enrollment and data collection are described in Fig. 1.

### Demographic data

**Fig. 2:**
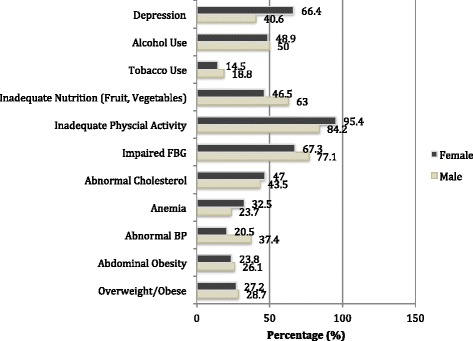
Sex-specific prevalence of CVD risk factors in adolescents of a Shantytown in Lima, Peru

The majority (56.4%) of the study population was female, and the median age of participants was 14 years of age (range 12−18 years). Seventy-four (26.9%) participants were in first grade, 26.5% in second grade, 22.6% in third grade, and 24% in fourth grade of secondary school in the 2015 school year. Average household size was six persons, and 38.2% of participants reported having visited a health clinic in the last year. Although the majority of participants denied knowledge of a family history (FH) of medical conditions, 16.9% reported a FH of diabetes, 13.6% reported hypertension, 23.2% reported high cholesterol, and 9.9% reported heart disease (Fig. 2).

### Anthropometric and clinical risk factor prevalence

Over one-quarter (27.8%) of adolescents were overweight or obese with no gender differences (28.7% males vs. 27.1% females; *p* = 0.78). The range of abdominal circumference was 61–109 cm (SD 9) for males, and 57–98 cm (SD 7) for females. Based on this circumference, 15.7% of males and 15.2% of females were at high risk of developing CVD, and 10.4% of males and 8.6% of females were at very high risk [29, 35]. Abdominal obesity was noted among 24.8% (26.1% males and 23.8% females) of adolescents (*p* = 0.67). Abnormal systolic or diastolic BP were detected more frequently in males than females; 37.4% of males were categorized as either pre-hypertensive (18.3%) or hypertensive (19.1%) and 20.5% of females as pre-hypertensive (11.9%) or hypertensive (8.6%) (*p* = 0.002). According to the capillary puncture data, 23.7% males and 32.5% of females had hemoglobin within mild (22.8% males, 25.8% females) or moderate (0.9% males, 6.6% females) anemia (*p* = 0.12). The lowest hemoglobin reading was 8.5 g/dL in a female participant. Nearly half of adolescents of both genders (45.5%) had an abnormal total cholesterol (43.5% males, 47.0% females) with a range of 151–257 mg/dL. A majority (71.0%) of adolescents presented impaired FBG levels (77.1% males, 67.3% females); 5.5% of whom had a FBG >126 mg/dL (8.6% males, 3.5% females).

### Behavioral and psychosocial risk factor prevalence

The most prevalent behavioral risk factor was inadequate physical activity (90.6%); more females (95.4%) denied engaging in intense daily physical activity than males (84.2%) (*p* = 0.002). Adolescents also reported high levels of sedentary behavior, with a daily average of 3.4 h of television (SD 3.6), 3.9 h of cellular phone use (SD 5.5) and 2.1 h of internet (SD 4.1), with an average daily screen time of 9.4 h. Daily consumption of vegetables was less frequent (32.1%) than fruits (42.3%) and 11.4% of adolescents reported “often or always” adding salt to their food.

Alcohol use was reported more frequently than tobacco use (49.4 vs. 16.3%) or marijuana use (4.4%). Of the 125 participants who reported having tried alcohol, the majority noted drinking with friends (39.8%) or family (30.9%); 69.9% endorsed drinking once annually; however, 18.6% reported drinking more than three times per month. The mean age for first-time alcohol experimentation was 11.9 years (SD 2.82) vs. 11.7 years (SD 2.53) for tobacco experimentation. Forty-two adolescents (17.1%) reported living or having lived with a family member who abused alcohol, and 14.2% reported having a family member who used tobacco. No major differences were noted by sex.

Depression within the last 2 weeks was prevalent in both genders (55.3%) with higher prevalence among females than males (66.4 vs. 40.6%; *p* < 0.001). Similarly, anhedonia was reported in 67.6% of females and 50% of males (*p* = 0.006). Females were more than twice as likely to report past or present thoughts of self-harm than males (37.9 vs. 15.7%); *p* < 0.001) and were nearly three times as likely to report a family history of mental illness as compared to their male counterparts (23.4 vs. 8.1%; *p* = 0.002). Females also reported more frequent physical abuse at home (31.4 vs. 25.7% of males) but less at school (5.4 vs. 10.4% of males). Six females and one male adolescent endorsed a history of sexual abuse.

In unadjusted logistic regression models, males were more than twice as likely to have hypertension (OR = 2.31, 95% CI = 1.34, 3.99, *p* = 0.002) and nearly twice as likely to have impaired fasting blood glucose (FBG) (OR = 1.96, 95% CI = 1.09, 3.52, *p* = 0.02). However, males were significantly less likely to be physically inactive (OR = 0.26, 95% CI = 0.10, 0.64, *p* = 0.002), be depressed (OR = 0.34, 95% CI = 0.20, 0.58, *p* < 0.001), or conduct self-harm behavior (OR = 0.31, 95% CI = 0.16, 0.57, *p* < 0.001). The relationships between clinical and behavioral risk factor and sex are described in Table 2.

**Table 2:** Association of clinical and behavioral risk factors by sex in adolescents of a Shantytown in Lima, Peru

	Overweight/obesity	High blood pressure	Anemia	High cholesterol	Impaired FBG	Inadequate physical activity
	Unadjusted OR, (95% CI)	Unadjusted OR, (95% CI)	Unadjusted OR, (95% CI)	Unadjusted OR, (95% CI)	Unadjusted OR, (95% CI)	Unadjusted OR, (95% CI)
Male sex	1.08 (0.63, 1.85)	2.31** (1.34, 3.99)	0.65 (0.37, 1.12)	0.87 (0.53, 1.41)	1.96* (1.09, 3.52)	0.26* (0.10, 0.64)
	Depression	Self-harm	Inadequate fruit intake	Inadequate vegetable intake	Tobacco use	Alcohol use
	Unadjusted OR, (95% CI)	Unadjusted OR, (95% CI)	Unadjusted OR, (95% CI)	Unadjusted OR, (95% CI)	Unadjusted OR, (95% CI)	Unadjusted OR, (95% CI)
Male sex	0.34*** (0.20, 0.58)	0.31*** (0.16, 0.57)	0.68 (0.41, 1.10)	1.13 (0.68, 1.88)	1.36 (0.70, 2.64)	1.04 (0.63, 1.73)

*p < 0.05; **p < 0.01; ***p < 0.001

The interrelationship between major CVD outcomes (overweight/obesity, high BP, abnormal FBG) with related risk factor exposures is described in Table 3. Overweight or obese adolescents were nearly twice as likely (PR = 1.98, 95% CI = 1.25–3.13, *p* = 0.004) to have high BP or an impaired FBG (PR = 1.74, 95% CI = 0.94–3.22, *p* = 0.08). High BP was also more often found among male adolescents (PR = 1.82, 95% CI = 0.94–1.29, *p* = 0.01) and in those with elevated cholesterol (PR = 1.57, 95% CI = 0.99–2.49, *p* = 0.05).

**Table 3:** Prevalence ratio for development of CVD risk factors among adolescents of a Shantytown in Lima, Peru

	Overweight/obese	High blood pressure	Impaired FBG
	PR (95% CI)	PR (95% CI)	PR (95% CI)
Age	0.92 (0.78–1.08)	1.10 (0.94–1.29)	0.95 (0.86–1.05)
Gender	1.06 (0.67–1.67)	1.82 (1.15–2.89)***	1.18 (0.89–1.55)
Overweight	NA	1.98 (1.25–3.13)*	1.17 (0.87–1.58)
High blood pressure	1.98 (1.25–3.13)*	NA	1.09 (0.81–1.48)
Anemia	1.00 (0.61–1.67)	0.44 (0.23–0.84)	1.06 (0.78–1.43)
High cholesterol	0.96 (0.61–1.53)	1.57 (0.99–2.49) ***	1.07 (0.81–1.42)
Impaired FBG	1.74 (0.94–3.22)**	1.32 (0.75–2.33)	NA
Inadequate physical activity	0.98 (0.45–2.14)	0.85 (0.41–1.76)	1.18 (0.71–1.98)
Depression	0.97 (0.60–1.56)	0.64 (0.40–1.04)	0.96 (0.71–1.29)
Self-harm	0.73 (0.41–1.33)	0.48 (0.24–0.93)	0.97 (0.70–1.35)
Inadequate fruit intake	1.29 (0.81–2.04)	1.03 (0.65–1.64)	1.04 (0.78–1.37)
Inadequate vegetable intake	0.94 (0.57–1.54)	1.07 (0.66–1.72)	0.98 (0.73–1.32)
Tobacco use	0.82 (0.42–1.60)	0.69 (0.32–1.44)	1.06 (0.73–1.55)
Alcohol Use	0.99 (0.62–1.59)	0.66 (0.40–1.08)	1.03 (0.77–1.40)

*p < 0.05; **p < 0.01; ***p < 0.001

## Discussion

Adolescence is a formative stage of development when poor health choices are developed and can form into lifelong habits [12]. Our findings suggest that many adolescents living in Lomas de Zapallal have risk factors (anthropometric, clinical, behavior, and psychosocial) that increase their likelihood of developing CVD as adults; this is supported by a cohort study of Dutch children followed longitudinally from 10–17 years of age that illustrated a pattern of adopting behaviors placing adolescents at risk for developing CVD (poor diet, physical inactivity, tobacco use) that worsened with increasing age and was highest in individuals from low-socioeconomic backgrounds [36]. Our present study highlights the importance of implementing risk prevention methods targeting this age group to address both physical and behavioral risk factors.

The observed prevalence of risk factors was higher than expected among our adolescent Peruvian population and varied by gender. Males and females of Lomas de Zapallal were comparably overweight or obese to adolescents living in developed countries but much more overweight than adolescents living in developing countries [37]. In addition, overweight or obese status was more prevalent in our male adolescent population compared to other Peruvian adolescent males <20 years old (23.8 vs. 16.6%). Childhood and adolescent obesity are driven by high caloric intake, physical inactivity, and sedentary behaviors [38] such as increased time watching TV, playing video games, or online activities [39]; behaviors that were highly prevalent in our population. According to data from the USA, adolescent obesity trends disproportionately affect youth of lower socio-economic backgrounds who have less access to safe spaces for physical activity and whose diets rely more heavily on processed foods [40].

As exemplified by our data, obesity was a key independent risk factor for future development of co-morbid hypertension, metabolic (abnormal fasting blood glucose), body image dissatisfaction [41], and other psychosocial diseases [14, 42]. Our adolescent population attending a public secondary school in peri-urban Lima had a higher risk for depression than another population of age-matched Peruvian adolescents attending a private school [41], especially among females. Depression during adolescence stimulates physiological systems (autonomic nervous system, fibrinogen, pro-inflammatory cytokines, neurohormones) correlated with both future development of CVD, as well as unhealthy behavioral coping strategies (substance use, inactivity, poor dietary choices) [6, 19]. A meta-analysis of modifiable risk factors associated with depression in adolescents showed a positive correlation between adolescent substance use (alcohol, tobacco, and marijuana use), dieting, early sexual activity, and overweight or obese status and a negative correlation with healthy diet, physical activity, adequate sleep, involvement with extracurricular activities, and good parental relationships [43]. This cycle of risk is complicated for our adolescent population living in a low-resource urban shantytown where a high prevalence of single-family homes, adult mental illness, and substance use limit access to positive role models [10].

### Limitations

Our biologic measures were imperfect. For instance, for the fasting blood glucose, some participants exerted themselves prior to participation and 31% did not fast prior to blood collection. A capillary puncture was used instead of a venous draw to collect cholesterol, glucose, and hemoglobin data. However, each point-of-care device was chosen for the high correlation with laboratory-based diagnostic assays [44, 45] and all health professionals received formal training on device operation prior to sample collection. Another limitation of our study is the small sample size, which reduces the power of our observations.

### Generalizability

Due to the unique socio-environmental factors of Lomas de Zapallal, the current data limit the generalizability of CVD risk factors to other Peruvian adolescents. It does, however, serve as an example of the increased risk for other youth living in peri-urban slum populations in Lima, Peru, as well as other impoverished areas of the world. Expansion of the study to include additional Peruvian secondary schools with varied socio-economic status could provide a more comprehensive picture of the CVD risk factors of adolescents living in South America.

## Conclusions

Our study provides a comprehensive snapshot of the CVD risk factor profile among adolescents living in a northern Lima shantytown population. When compared to other local and global adolescent populations, adolescents living in Lomas de Zapallal had comparable or higher prevalence of risk factors associated with development of CVD [21, 46–49]. To decrease these risk factors for this and future generations of adolescents, integrative community-based programs should consider and address the multiple facets of adolescent behaviors, including physical, emotional, environmental, and social factors identified in our study population.

## Abbreviations

AHA: American Heart Association
BMI: Body mass index
BP: Blood pressure
CDC: Center for Disease Control
CVD: Cardiovascular disease
FBG: Fasting blood glucose
NHANES: National Health and Nutrition Examination Survey
NIH: National Institute of Health
POC: Point-of-care
WC: Waist circumference
WHO: World Health Organization
YRBSS: Youth Risk Behavior Surveillance System
